# Gypsum-DL: an open-source program for preparing small-molecule libraries for structure-based virtual screening

**DOI:** 10.1186/s13321-019-0358-3

**Published:** 2019-05-24

**Authors:** Patrick J. Ropp, Jacob O. Spiegel, Jennifer L. Walker, Harrison Green, Guillermo A. Morales, Katherine A. Milliken, John J. Ringe, Jacob D. Durrant

**Affiliations:** 1 Department of Biological Sciences, University of Pittsburgh, Pittsburgh, PA 15260 USA; 2 Department of Chemistry and Chemical Biology, Indiana University-Purdue University Indianapolis, Indianapolis, IN 46202 USA; 3 Innoventyx, LLC, Oro Valley, AZ 85737 USA

**Keywords:** Small-molecule libraries, Virtual screening, Chemical biology, Computer-aided drug discovery, Computational biology, 3D structure generation

## Abstract

Computational techniques such as structure-based virtual screening require carefully prepared 3D models of potential small-molecule ligands. Though powerful, existing commercial programs for virtual-library preparation have restrictive and/or expensive licenses. Freely available alternatives, though often effective, do not fully account for all possible ionization, tautomeric, and ring-conformational variants. We here present Gypsum-DL, a free, robust open-source program that addresses these challenges. As input, Gypsum-DL accepts virtual compound libraries in SMILES or flat SDF formats. For each molecule in the virtual library, it enumerates appropriate ionization, tautomeric, chiral, cis/trans isomeric, and ring-conformational forms. As output, Gypsum-DL produces an SDF file containing each molecular form, with 3D coordinates assigned. To demonstrate its utility, we processed 1558 molecules taken from the NCI Diversity Set VI and 56,608 molecules taken from a Distributed Drug Discovery (D3) combinatorial virtual library. We also used 4463 high-quality protein–ligand complexes from the PDBBind database to show that Gypsum-DL processing can improve virtual-screening pose prediction. Gypsum-DL is available free of charge under the terms of the Apache License, Version 2.0.
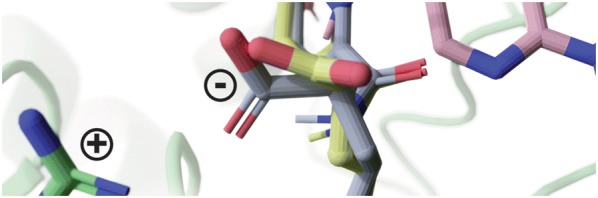

## Introduction

Structure-based virtual screening (VS) is a powerful tool for pharmacological and basic-science research [1, 2]. In a successful VS campaign, a docking program poses small-molecule models within a protein binding pocket, and a scoring function estimates binding affinities. Experimentalists then test the top-scoring compounds to verify binding. Hit rates are often better than those obtained through high-throughput screening alone [2].

The first and foundational step in a VS workflow is pose prediction. Accurate prediction depends on high-quality 3D models of both protein receptor(s) and potential small-molecule ligands. Small-molecule databases often store compounds in formats that include only atom-type and bond information (e.g., SMILES). Furthermore, database entries typically describe only one ionization or tautomeric state per molecule, and they may lack information about chirality and cis/trans isomerization.

Though effective, available commercial and open-source programs for processing and converting these simple representations into fully enumerated 3D models have their drawbacks. Commercial programs such as OpenEye’s OMEGA/QUACPAC [3, 4] and Schrödinger’s LigPrep (Schrödinger, LLC) have restrictive licenses and can be expensive. While OpenEye does offer a free academic license, that license imposes substantial commercialization and intellectual-property restrictions. License eligibility is also regularly re-evaluated, making long-term access uncertain. And workflows that incorporate commercial tools cannot typically be freely distributed.

Free alternatives include Frog2 [5] and Balloon [6, 7]. Frog2 [5] is an open-source, web-based program that requires no installation. Users must first upload their compounds in SMILES format to the RPBS Web portal (http://bioserv.rpbs.univ-paris-diderot.fr/services/Frog2/) [8, 9]. The Frog2 server then assigns 3D coordinates and provides a downloadable file containing the results. In contrast, Balloon [6, 7] is a command-line program that can be easily and freely incorporated into larger workflows. Though Balloon is free, the source code is not publicly available, and the program does not account for alternate chiral and cis/trans isomeric forms. Additionally, Frog2 and Balloon ignore alternate ionization and tautomeric forms; sometimes miss low-energy, non-aromatic ring conformations; and generate excess rotamers beyond those needed for flexible-ligand docking.

The popular open-source cheminformatics package Open Babel [10] also includes several executable files that can perform key small-molecule preparation steps. For example, the *obabel* executable accepts a -*p* (pH) parameter that ionizes molecules as appropriate for a user-specified pH. The *obabel* –*gen3D* (generate 3D coordinates) parameter also converts molecular representations to 3D models that can be further optimized with *obminimize*. But it is difficult to generate alternate tautomeric, chiral, and cis/trans isomeric forms using Open Babel’s command-line interface. Advanced users/programmers must implement these features separately using Open Babel’s programming API. Open Babel is also released under a copyleft license (GNU General Public License, version 2), which requires that any derivate works also be copyleft.

To address the limitations of existing commercial and open-source packages, we here present Gypsum-DL, a free, open-source program for preparing small-molecule libraries. Beyond simply assigning 3D coordinates, Gypsum-DL outputs molecular models with varying ionization, tautomeric, and isomeric states. Protein binding pockets often stabilize these alternate forms, even if their prevalence is low in bulk solution. Gypsum-DL also generates models with alternate non-aromatic ring conformations. Considering alternate ring conformations is critical given that most flexible-ligand docking programs (e.g., AutoDock Vina [12]) do not account for all possible ligand ring geometries during the docking process itself.

We use 4463 high-quality protein–ligand complexes from the PDBBind database (http://www.pdbbind.org.cn/) [13, 14] to show that Gypsum-DL processing can improve VS pose prediction. To further show utility, we also use Gypsum-DL to process two virtual molecular libraries: (1) the NCI Diversity Set VI, a set of freely available compounds provided by the National Cancer Institute (1558 molecules); and N-acylated unnatural amino acids enumerated using the accessible chemical reaction schemes developed by the Distributed Drug Discovery (D3) initiative (56,608 molecules) [15–18]. These virtual libraries are available free of charge for use in VS projects.

Gypsum-DL will be a helpful tool for those engaged in both basic-science and drug-discovery research. A copy is available at http://durrantlab.com/gypsum-dl/, released under the terms of the Apache License, Version 2.0.

## Implementation

### The Gypsum-DL algorithm

**Fig. 1:**
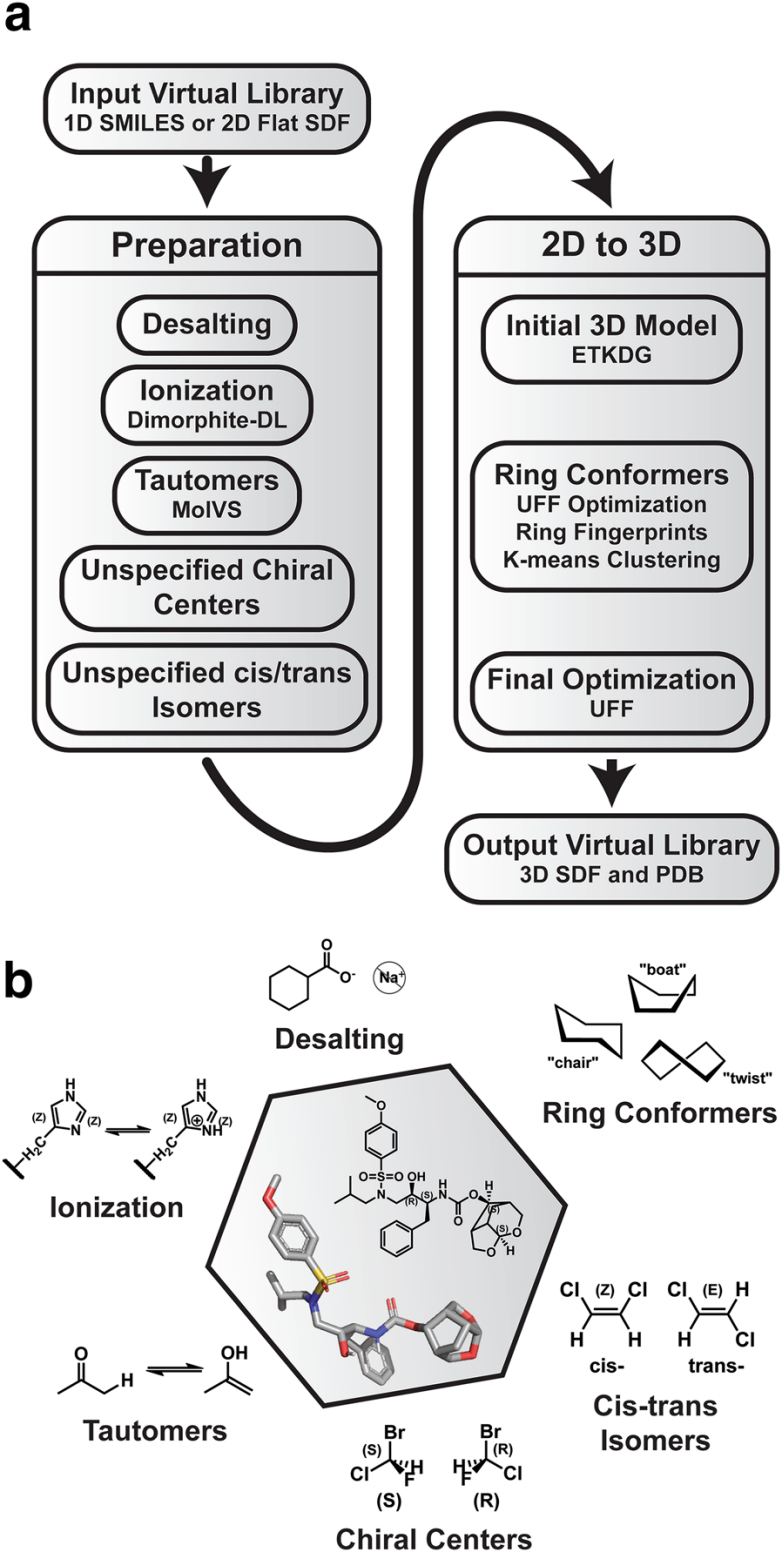
The Gypsum workflow. **a** Gypsum prepares a virtual small-molecule library by desalting the input compounds and considering alternate ionization, tautomeric, chiral, and cis/trans isomeric states. It then converts all variants to 3D, accounting for alternate ring conformations where appropriate. **b** Illustrative examples of each Gypsum step

Gypsum-DL uses RDKit (http://www.rdkit.org), MolVS 0.1.1 (https://molvs.readthedocs.io), and Dimorphite-DL 1.0 [11] to convert small-molecule representations (SMILES strings or flat SDF files) into 3D models (Fig. 1) [19]. Each output SDF file includes fields that describe the steps used to generate the corresponding model. Gypsum-DL also leverages multiple processors, if available, to speed the conversion of large virtual libraries. Command-line flags allow the user to precisely control all aspects of the program, though the default parameters should serve most use cases.

#### Desalting

Gypsum-DL first removes any salts present in the user-specified virtual compound library. Molecular representations (e.g., SMILES) often include the primary molecule together with accompanying counterions. Gypsum-DL retains only the largest fragment, the presumed compound of interest.

#### Ionization

Gypsum-DL uses the Dimorphite-DL 1.0 algorithm [11] to generate models with different ionization states. It considers a user-defined range of pH values (6.4–8.4 by default) rather than a single (e.g., physiological) pH. Separate models are created for each identified state.

Given the computational demands of high-throughput VS, it is important to limit the number of ionization forms considered. To eliminate highly charged forms that are unlikely to be physiologically relevant, Gypsum-DL first identifies the generated ionization form with a formal charge that is closest to zero. It eliminates any additional ionization forms whose formal charges deviate from that baseline by 3 *e* or more.

#### Tautomeric forms

Many compounds readily interconvert between tautomeric states as protons and electrons shift among atoms. Gypsum-DL uses MolVS 0.1.1 to enumerate all possible tautomers. It discards tautomers that alter the number of aromatic rings (i.e., by breaking ring aromaticity) or the number of chiral centers. Separate models are created for each identified tautomeric form.

MolVS occasionally produces particularly improbable tautomeric forms. Gypsum-DL maintains a list of substructures associated with these forms and automatically eliminates any matching models. For example, though Gypsum-DL does consider keto–enol tautomerism, it does not permit enol forms that result in terminal alkenes. It also eliminates compounds with geminal vinyl diols, which are improbable tautomers of carboxylic acids. Any form with a carbanion is also eliminated, as are tautomers that disrupt existing aromaticity.

#### Unspecified chiral centers and cis/trans double-bond isomerization

Many virtual-library databases do not fully specify all compound chiral centers. Gypsum-DL thoroughly generates alternate chiral species by varying each of the unspecified chiral centers in each input molecule. Specifically defined chiral centers remain unchanged. Similarly, virtual-library databases often include compounds with unspecified double-bond isomerization. Gypsum-DL systematically and thoroughly generates alternate cis/trans isomers as needed.

We note that MolVS removes double-bond cis/trans stereochemistry if any derived tautomeric form changes the double bond to a single bond. Some compounds may thus end up with unspecified double bonds, even if the input molecular representation explicitly specifies isomerization. This behavior is intentional, though it may surprise some users. Gypsum-DL enumerates both the cis and trans isomers in such cases.

#### Alternate conformations of non-aromatic rings

To sample different small-molecule conformers, flexible-ligand docking programs permit virtual rotations about single bonds during the docking process. But they often treat rings as rigid, even if those rings include single bonds. Transitions between different ring conformations (e.g., the boat and chair conformations of cyclohexane) are thus ignored. Gypsum-DL addresses this shortcoming by generating separate models with distinct low-energy ring conformations.

**Fig. 2:**
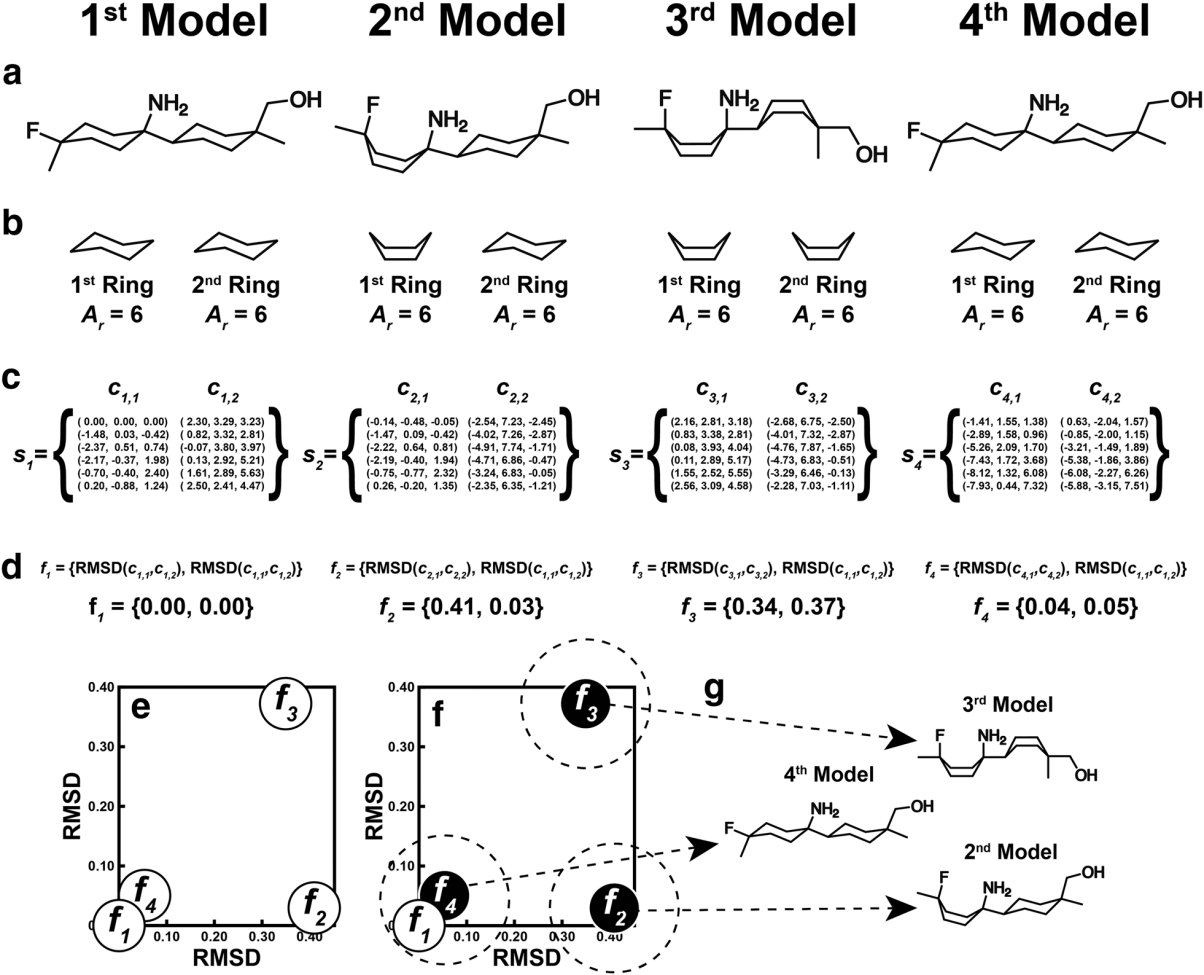
A schematic of the Gypsum-DL algorithm for generating ring-conformational forms. **a** Create multiple 3D variants using ETKDG and UFF optimization. **b** Extract the rings. **c** Collect the coordinates of the ring atoms. **d** Construct ring fingerprints by calculating the RMSD between each ring and the corresponding ring of the first model. **e**, **f** Use *k*-means clustering to identify unique ring fingerprints. The small circles on the graphs represent fingerprints, the larger dashed circles represent clusters, and the black circles represent the most central fingerprint of each cluster. **g** The central fingerprints correspond to geometrically unique models

Gypsum-DL first generates multiple 3D models of each input molecule using the Experimental Torsion with Knowledge Distance Geometry (ETKDG) method (version 2 if available, or version 1 otherwise) [20]. These initial models are then optimized using the Universal Force Field (UFF) [21]. Though this optimization step is computationally expensive, it encourages 3D ring conformers that closely correspond to discrete energy minima. For a given compound with *R* non-aromatic rings, there are thus *M* optimized 3D models (see Fig. 2a, where *R* = 2 and *M *= 4).

For reference, we assign an index, *m*, to each of the *M* models. We also assign an index, *r*, to each of the *R* non-aromatic rings, and we say that the *r*th ring contains *A*_*r*_ atoms (Fig. 2b). There are thus *M* total conformations of the *r*th ring, one corresponding to each modeled compound. The *r*th ring of the *m*th model refers to a specific ring. To describe the geometry of that ring, Gypsum-DL places the 3D coordinates, (*a*_*x*_, *a*_*y*_, *a*_*z*_), of its *A*_*r*_ constituent atoms into an ordered list, *c*_*m*,*r*_ (Fig. 2c), where

$$c_{m,r} = \left\{ {\left( {a_{x} ,a_{y} ,a_{z} } \right) |a \in {\mathbb{N}},\text{ }a \le A_{r} } \right\}$$

To describe the collective ring-conformational geometry of the *m*th model, Gypsum-DL collects the geometries of the *R* associated rings, *c*_*m*,*r*_, into another ordered list, *s*_*m*_ (Fig. 2c):

$$s_{m} = \left\{ {c_{m,r} |r \in {\mathbb{N}},\text{ }r \le R} \right\}$$

To quantify how much a given 3D models’ ring conformations collectively differ from those of the first model, Gypsum-DL generates an *R*-dimensional “ring-conformation fingerprint,” *f*_*m*_ (Fig. 2d), for each of the *s*_*m*_ lists:

$$f_{m} = \left\{ {RMSD\left( {c_{m,r} , c_{1,r} } \right) |r \in {\mathbb{N}},\text{ }r \le R} \right\}$$

where the function RMSD(*c*_1_, *c*_2_) is the minimum root-mean-square deviation (RMSD) between coordinate set *c*_1_ and coordinate set *c*_2_ when *c*_1_ is allowed to freely rotate and translate.

The first fingerprint (*f*_1_) is thus an *R*-dimensional zero vector because the conformations of the first-model rings are identical to themselves. Subsequent *f*_*m*_ are *R*-dimensional vectors whose entries represent the extent to which the conformation of the corresponding ring differs from that of the same ring in the first model.

Among the *M* models generated, some may have very similar ring conformational states. To eliminate this redundancy, Gypsum-DL uses *k*-means clustering [22] to cluster the set of all *f*_*m*_ into at most *max_variants_per_compound* groups, where *max_variants_per_compound* is a user parameter (default: 5; Fig. 2e, f). Only the 3D models corresponding to the most central *f*_*m*_ of each cluster are retained (Fig. 2g).

#### Controlling the combinatorial explosion

Gypsum-DL accounts for alternate ionization, tautomeric, chiral, isomeric, and ring-conformational states. From an algorithmic perspective, each of these five states is independent. It is thus possible to generate an intractable number of models per input molecule. For example, consider a molecule with two variants for each state. Accounting for all possible forms would require 2^5^ = 32 models per molecule. Performing a VS of 10,000 compounds would thus require 320,000 separate dockings.

To prevent this combinatorial explosion, after each step Gypsum-DL prunes the growing set of enumerated forms associated with each input molecule. It first randomly selects *m* x *t* variants from all the forms generated, where *m* is the *max_variants_per_compound* user parameter (default: 5) and *t* is the *thoroughness* user parameter (a scaling factor, default: 3). It then uses ETKDG [20] to generate a 3D conformer for each of the selected *m* × *t* variants. The energies of these conformers are evaluated using the UFF [21]. To reduce computational cost, Gypsum-DL generally performs this evaluation without geometry optimization (except for compounds with non-aromatic rings, see above). It ultimately retains only the *m* compounds from the *m* x *t* variants with the best predicted energies.

#### Final geometry optimization and output

As a final step, Gypsum-DL uses the UFF to optimize the geometries of any remaining compounds that have not already been optimized (i.e., any compound that was not already optimized in the ring-conformation step). It saves the resulting 3D models to SDF file(s) in a user-specified directory. The user can also instruct the program to additionally save conformers in the PDB format. Optional output to an HTML file allows the user to quickly visualize the generated structures in 2D.

### Gypsum-DL and VS pose prediction

To assess the impact of Gypsum-DL processing on VS pose prediction, we compiled a benchmark library of protein–ligand complexes. We first downloaded the 4463 high-quality complexes included in PDBBind refined set [13, 14]. We removed those complexes with ligands that had molecular weights greater than 500 Daltons, contained amino acids, contained multiple residues (e.g., peptides), included improper atom names (e.g., “furan”), and/or had ligand files that did not match the corresponding entries in the Protein Data Bank (https://www.rcsb.org/) [23]. After filtering, 3177 complexes containing 2438 unique ligands remained.

For each ligand, we downloaded the corresponding SMILES string from the Protein Data Bank [23]. We neutralized the charge of each SMILES representation to the extent possible and removed all information about chirality and cis/trans isomerism. We converted these processed SMILES strings to 3D models using both Open Babel 2.3.2 and a late-stage beta version of Gypsum-DL that did not differ substantially from the final published version. For Open Babel, we used only the -*d* (delete hydrogens), -*h* (add hydrogens), and –*gen3D* (generate 3D coordinates) flags, in that order, to standardize hydrogen atoms and generate 3D coordinates. For Gypsum-DL, we used the following ligand-processing parameters: *min_ph* 6.4, *max_ph* 8.4, *pka_precision* 1.0, *thoroughness* 3, and *max_variants_per_compound* 5. We then converted all protein-receptor and small-molecule models to the PDBQT format using MGLTools 1.5.6 [24].

For each complex, we defined a docking box that entirely encompassed the corresponding crystallographic ligand, with 5 Å margins in all directions. We then used AutoDock Vina 1.1.2 [12] to dock the Open-Babel and Gypsum-DL small-molecule models into the corresponding docking boxes. The default AutoDock Vina parameters were used, except we increased the exhaustiveness parameter to 100.

To judge pose-prediction accuracy, we first used our Scoria Python library [25] to remove the hydrogen atoms from all docked compounds. We then used *obrms* [10], an Open-Babel utility program, to calculate the RMSDs between the non-hydrogen atom positions of each top-scoring Vina pose and those of the corresponding crystallographic ligand. The *obrms* approach accounts for equivalent moiety conformations (e.g., symmetric ring flips) by considering atom connectivity. We discarded an additional 26 protein–ligand complexes because *obrms* determined inconsistent connectivities for the crystallographic pose versus the docked Open-Babel and/or Gypsum-DL poses. The size of the final benchmark library of protein–ligand complexes was thus 3151.

Unlike Open Babel, Gypsum-DL often generates multiple variants of each compound with differing ionization, tautomeric, chiral, cis/trans isomeric, and ring-conformational states. For comparison purposes, we selected the Gypsum-DL variant with the lowest RMSD to the crystallographic pose.

### Enumerating the distributed drug discovery (D3) library for Gypsum-DL testing

To test Gypsum-DL’s ability to process a large virtual molecular library, we enumerated 56,608 N-acylated unnatural amino acids. We first used ChemDraw Ultra 12.0 (CambridgeSoft, 2010) to create 2D representations of the 84 alkyl-halide, 16 Michael-acceptor, and 100 carboxylic-acid building blocks described in Ref. [16]. Some building blocks were racemic, so we expanded this initial set to include all associated enantiomers. We then used MarvinSketch 16.6.13, 2016, ChemAxon (http://www.chemaxon.com) to create the multi-step reaction schemes required to enumerate a 2D virtual library from these building blocks.

**Fig. 3:**
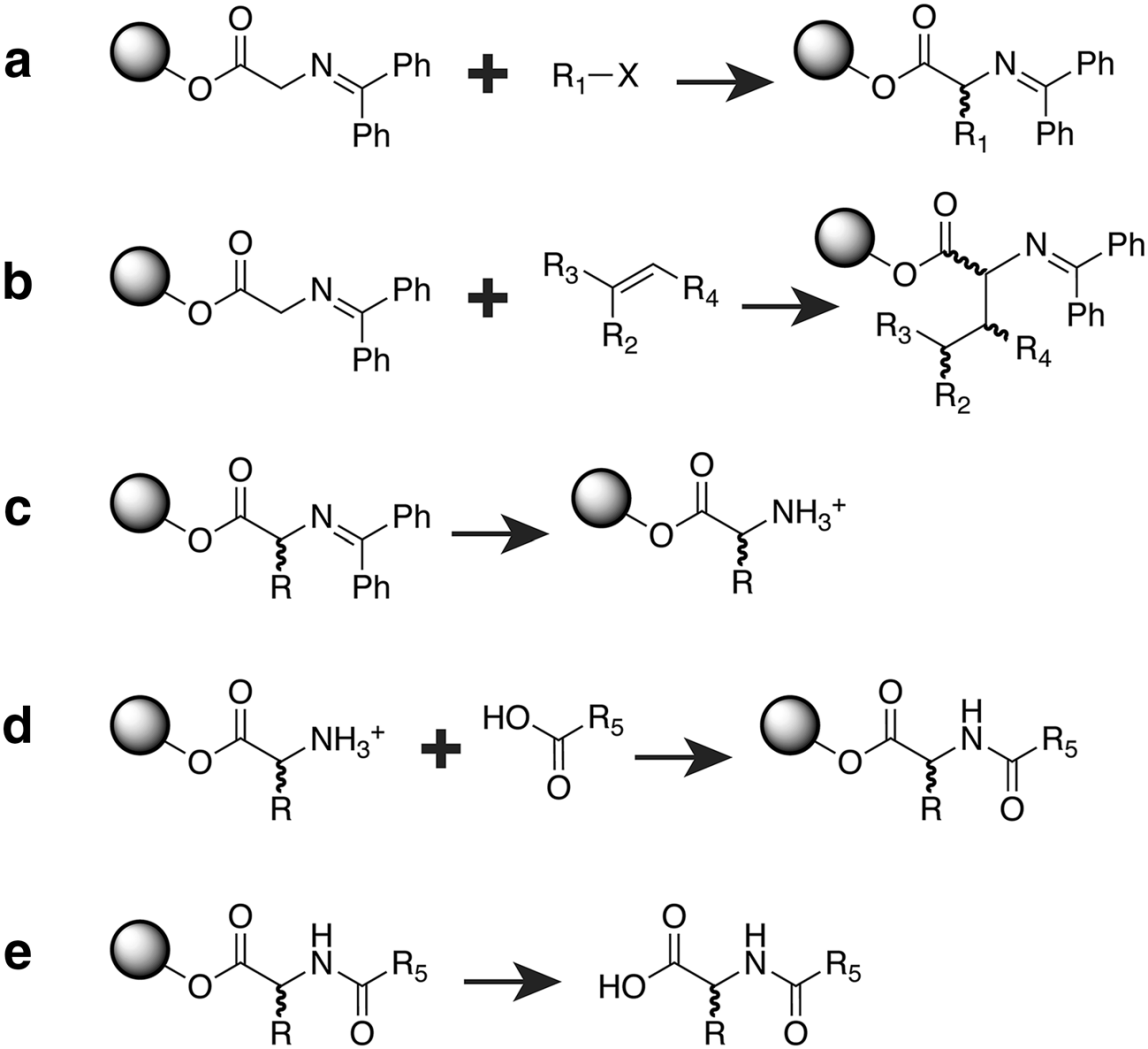
Simplified representations of the reaction schemes used to enumerate the D3 library. The spheres represent the bound polymer. **a** Alkylation of the polymer-bound benzophenone-imine glycine using the alkyl-halide building blocks. **b** Alkylation using the Michael-acceptor building blocks. **c** Deprotecting the benzophenone protecting group. **d** Acylation using the carboxylic-acid building blocks. **e** Cleaving the polymer

A detailed description of the reactions has been published previously [16]. We selected them in part because they are central to the highly successful undergraduate curriculum developed by the Distributed Drug Discovery (D3) initiative [16]. In brief, we first created reaction schemes to alkylate polymer-bound benzophenone-imine glycine at the carbonyl ɑ-carbon. For alkylation using alkyl halides, we created two reaction schemes to generate products with (*S*) and (*R*) stereochemistry, respectively (Fig. 3a). For alkylation using Michael acceptors, we created eight reaction schemes to enumerate all possible diastereomers (Fig. 3b). We used Reactor 16.6.13, 2016, ChemAxon (http://www.chemaxon.com) to apply these reaction schemes to our library of alkyl-halide and Michael-acceptor building blocks.

We next created a reaction scheme to deprotect the benzophenone protecting groups of the alkyl-halide- and Michael-reaction products, yielding amino-free intermediates (Fig. 3c). These intermediates were then subjected to an acylation scheme (Fig. 3d), which reacted the free amino groups with each of our carboxylic-acid building blocks. This reaction added another point of diversity, ultimately yielding polymer-bound alkylated-acylated glycine products. An additional reaction scheme served to cleave the polymer (Fig. 3e). We also used reaction schemes to remove additional protecting groups (e.g., Fmoc, tert-butyl, Boc), which the final products had inherited from some of the original building blocks (not shown).

### Using Gypsum-DL to process the D3 and NCI molecular libraries

We used Gypsum-DL to process both the enumerated D3 library and the NCI Diversity Set VI, a set of freely available compounds provided by the National Cancer Institute. We used the following Gypsum-DL parameters to process these libraries: *min_ph* 7.4, *max_ph* 7.4, *pka_precision* 1.0, *thoroughness* 3, and *max_variants_per_compound* 5. In the case of the D3 library, we additionally set the *skip_making_tautomers* parameter to true. Both libraries are available free of charge in the SDF format from http://durrantlab.com/gypsum-dl/ for use in VS projects.

## Results and discussion

### Installation

Gypsum-DL is an open-source Python program. It requires the third-party Python libraries RDKit, NumPy [26], and SciPy [27], which must be installed separately. To ease installation, we recommend the popular Anaconda Python platform with its convenient *conda* package manager. Users who wish to run Gypsum-DL using the Message Passing Interface (MPI) standard must also install the mpi4py package [28–30]. Gypsum-DL also relies on the MolVS library (MIT License). We have included a copy of MolVS 0.1.1 with the Gypsum-DL source code, so no additional installation is needed.

### Platform testing

We have tested Gypsum-DL on several operating systems, using several versions of Python, RDKit, NumPy, SciPy, and mpi4py (Table 1). We expect it will run in many other environments as well. We note that the multiprocessing feature is not available on Microsoft Windows.

**Table 1:** Computational environments used for Gypsum-DL testing

Operating system	Python	RDKit	NumPy	SciPy	Mpi4py
macOS Mojave 10.14.1	2.7.13	2016.09.2	1.11.3	0.19.0	N/A
macOS Mojave 10.14.1	3.6.3	2018.03.4	1.13.1	0.19.1	N/A
Ubuntu 18.04.1 LTS	3.7.1	2018.03.1	1.15.4	1.0.0	N/A
Ubuntu 18.04.1 LTS	3.6.6	2018.03.1	1.15.4	1.0.0	N/A
Ubuntu 18.04.1 LTS	2.7.1	2018.03.1	1.15.4	1.0.0	N/A
Windows 10 (serial mode)	3.7.1	2018.03.1	1.15.4	1.0.0	N/A
Windows 10 (serial mode)	2.7.1	2018.03.1	1.15.4	1.0.0	N/A
Red Hat Enterprise Linux Server 7.3	3.5.3	2018.03.1	1.12.1	1.0.0	N/A
Red Hat Enterprise Linux Server 7.3	2.7.13	2018.03.1	1.12.1	1.0.0	3.0.1

### Benchmarks

Gypsum-DL can take advantage of multiple processors, if available. The user-defined *job_manager* parameter determines whether the program runs in “serial,” “multiprocessing,” or “mpi” mode. In serial mode, Gypsum-DL uses only one processor to prepare each small molecule sequentially. This mode is ideal when processing only a few compounds or when using Gypsum-DL in low-resource environments. It is also the only mode available on the Windows operating system.

**Fig. 4:**
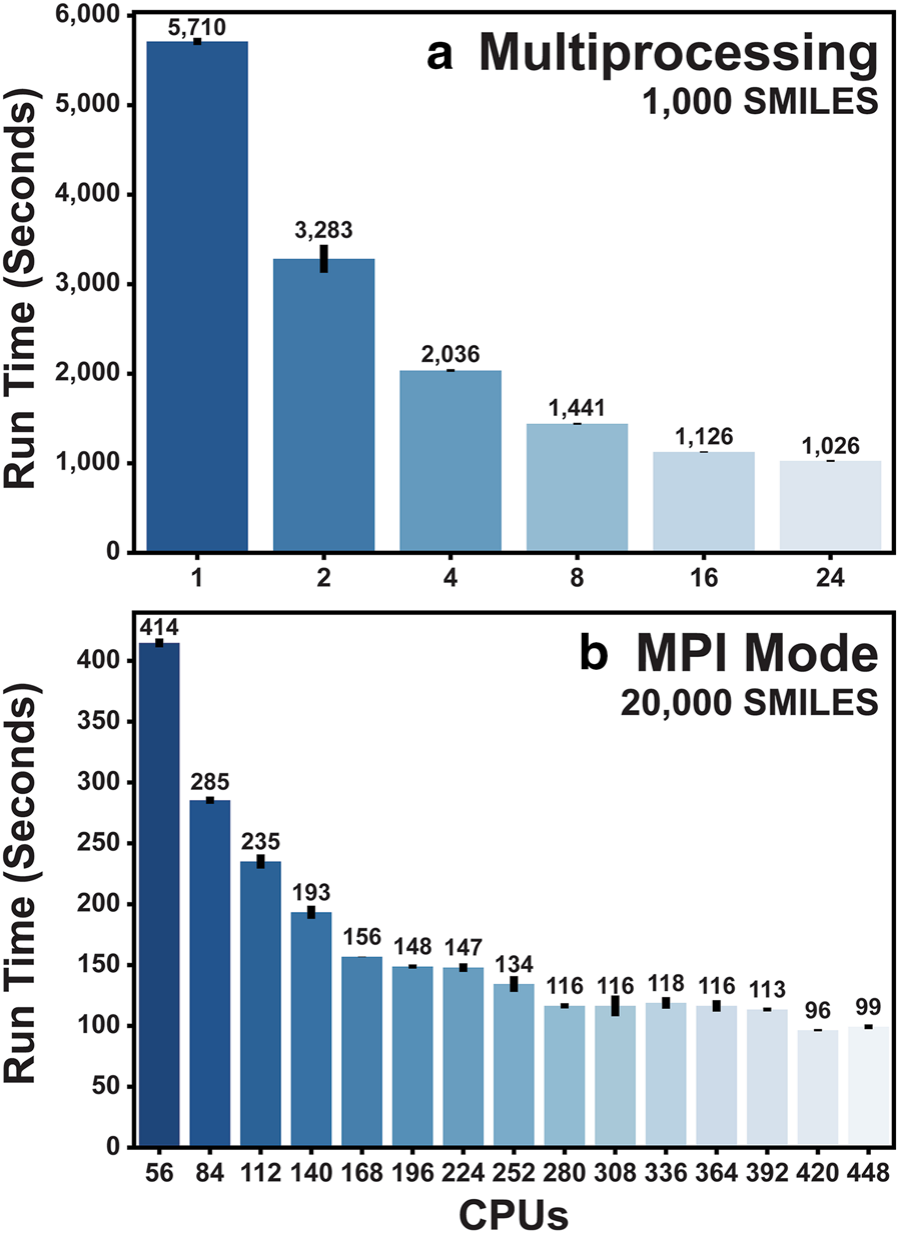
Gypsum-DL benchmarks. **a** Run times on a single compute node, using multiprocessing mode (1000 input SMILES strings). **b** Run times on multiple compute nodes, using mpi mode (20,000 input SMILES strings). All benchmarks were performed in triplicate. Errors bars represent standard deviations

In multiprocessing mode, Gypsum-DL uses multiple processors on the same computer to speed small-molecule preparation. Its dynamic load-balancing approach distributes small-molecule representations (e.g., SMILES strings) to various processors as they become available. Running in parallel, each processor independently prepares its assigned representations. Figure 4a shows benchmark run times performed on a 24-core Skylake processor using a late-stage beta version of Gypsum-DL that did not differ substantially from our final published version.

In mpi mode, Gypsum-DL distributes small-molecule preparation across multiple computers. Its static load-balancing approach splits the array of input small molecules into chunks that can each be handled concurrently on a different computer (i.e., node). This mode is ideal for use on high-performance computing clusters, where separate computers are networked together to enable calculations on a much larger scale. To leverage this setup, Gypsum-DL uses the Message Passing Interface (MPI) to control parallel communications between nodes. The user must separately install the mpi4py Python package [28–30] to use Gypsum-DL in mpi mode. We benchmarked the same beta version of Gypsum-DL on a computing cluster provided by the University of Pittsburgh’s Center for Research Computing (CRC, Fig. 4b). The CRC provides MPI-enabled compute nodes with 28-core Broadwell Processors, networked using Intel’s Omni-Path communication architecture. Note that the benchmarks shown in Fig. 4b were run on 20,000 input SMILES strings, vs. 1000 in Fig. 4a.

### Comments on scalability

In theory, processing an entire virtual library should be embarrassingly parallel. But in practice two factors prevent perfectly linear scalability. First, in mpi mode Gypsum-DL uses static rather than dynamic load balancing. It assigns each input representation (e.g., SMILES string) to a processor before execution begins. If the number of inputs is divisible by the number of processors, each processor is tasked with handling the same number of inputs. Otherwise, Gypsum-DL distributes the inputs as evenly as possible. Each processor then independently and concurrently prepares its portion of the input virtual library, without requiring synchronization or memory sharing. Once all processors have finished, the main process collects the results. Static load balancing minimizes the required communication between nodes, but it can lead to computational inefficiency. If by random chance a given processor is assigned many time-consuming molecular representations, other processors may run idle while waiting for it to finish. Increasing the number of representations assigned to each processor can reduce the chances of highly unbalanced assignments.

Second, in both multiprocessing and mpi mode, some tasks cannot be parallelized. For example, the main process must send input data to each processor/node and collect the results when finished. Furthermore, Gypsum-DL also spawns a separate Python interpreter on each processor to handle the assigned input. The fixed time required to start and shutdown each interpreter also impacts scalability. Increasing the time spent processing molecular representations relative to the communication/startup/shutdown times (again, by increasing the number of representations assigned to each processor) thus improves scaling.

In summary, using more processors can drastically reduce the total run time (Fig. 4). But as the input data is divided among more and more processors, the number of molecular representations handled per processor begins to drop. As with most large-scale parallel calculations, users must strike a balance between short run times and computational efficiency.

### Gypsum-DL improves pose-prediction accuracy

To test Gypsum-DL’s impact on the accuracy of VS pose prediction, we considered 3151 protein–ligand complexes taken from the PDBBind database [13, 14]. Both Gypsum-DL and Open Babel 2.3.2 were separately used to prepare 3D models of the 3151 ligands from the corresponding SMILES strings. In the case of Open Babel, we intentionally generated electrically neutral models (i.e., we omitted the Open-Babel -*p* flag) so as to better judge the impact of Gypsum-DL’s ionization feature on pose accuracy [11]. We docked both the Gypsum-DL-prepared and Open-Babel-prepared molecules into their corresponding protein receptors using AutoDock Vina 1.1.2 [12].

When we used Gypsum-DL, 71.4% of the 3151 ligands had RMSDs from the crystallographic pose that were less than 3.0 Å (mean: 2.37 Å; standard deviation: 2.03 Å). The same was true of 53.0% of the Open-Babel-processed molecules (mean: 3.40 Å; standard deviation: 2.51 Å). An F-test led us to reject the hypothesis that the variances of the Gypsum-DL and Open-Babel RMSDs were equal (*p* = 0.00). A subsequent two-tailed *t* test (assuming unequal variances) led us to reject the hypothesis that the Gypsum-DL and Open-Babel RMSDs had the same mean (*p* = 0.00). These results suggest that accounting for multiple ionization, tautomeric, chiral, cis/trans isomeric, and ring-conformational forms can improve pose-prediction accuracy.

**Fig. 5:**
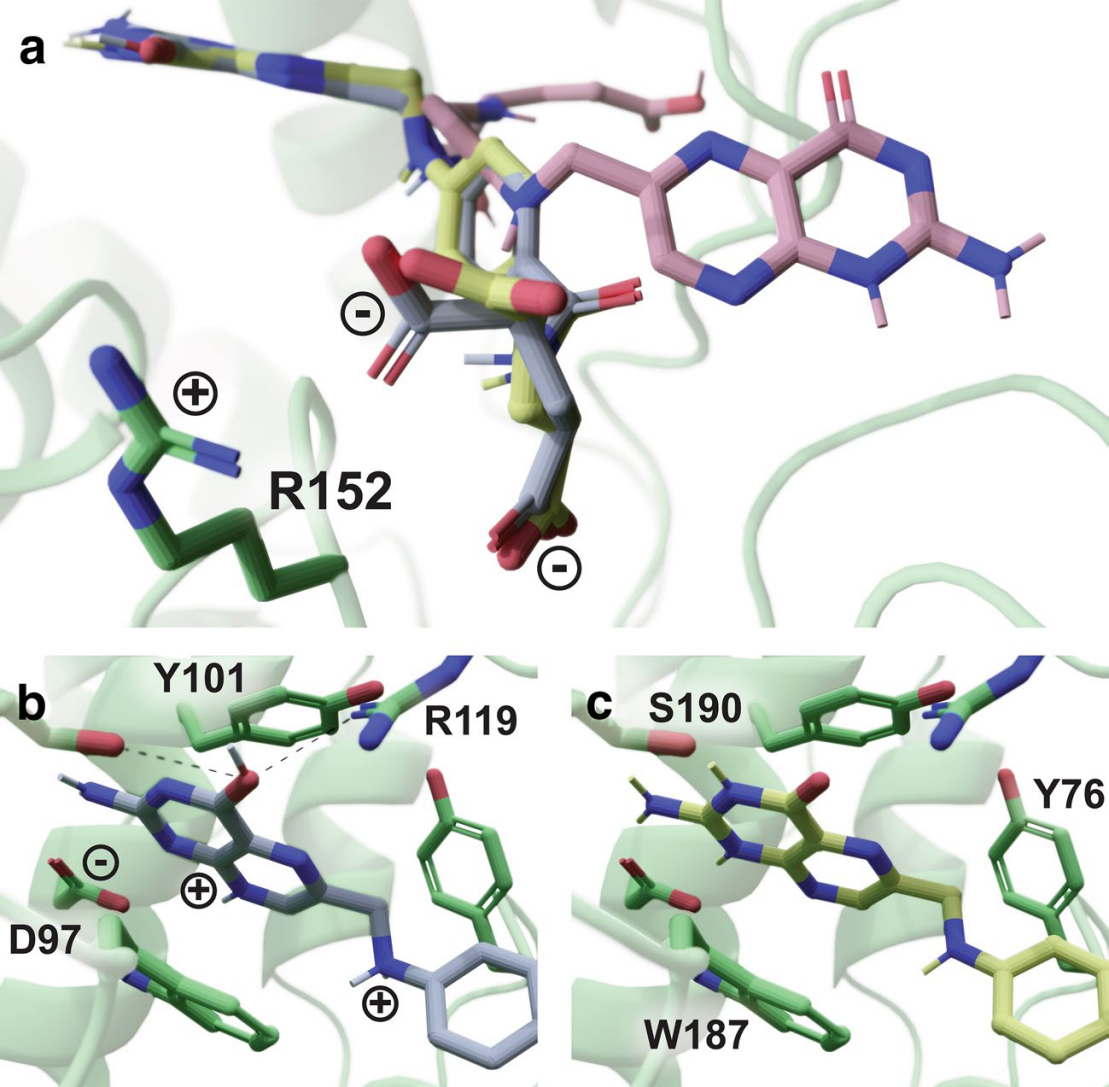
An illustration of the crystallographic and docked poses of folic acid bound to the human folate receptor beta. The carbon atoms of the protein, the crystallographic ligand, the docked Gypsum-DL compound, and the docked Open-Babel compound are shown in green, yellow, gray, and pink, respectively. **a** The region of the pocket near R152. **b** The Gypsum-DL-prepared compound forms additional interactions with other protein residues (in green). Possible hydrogen bonds are shown as dotted lines. **c** The crystallographic pose, shown for reference. Image generated using BlendMol [33]

As an example of the advantages of Gypsum-DL processing, consider folic acid bound to the human folate receptor beta (PDB ID: 4KMZ [31]; Fig. 5a). In this test case, the RMSD between the Gypsum-DL-prepared and crystallographic poses was only 0.76 Å (Fig. 4b, c). In contrast, the RMSD between the Open-Babel-prepared and crystallographic poses was 11.42 Å (Fig. 5a). Visual inspection of the docked molecules, together with structural analysis using BINANA 1.2.0, a program that automates the detection of key protein/ligand interactions [32], provides insight into why Gypsum-DL performed better. Gypsum-DL deprotonated the two folate carboxylate groups, allowing them to form strong electrostatic interactions with R152. In contrast, we did not instruct Open Babel to consider pH, so it protonated these carboxylate groups (Fig. 5a).

The input SMILES string represented folate in the favored 2-aminopteridin-4(1*H*)-one (keto) form. Open Babel does not generate alternate tautomeric states and so used this same form. Interestingly, Gypsum-DL selected the enol tautomer, 2-aminopteridin-4-ol. And yet in the accurate Gypsum-DL pose, the enol hydroxyl group may form hydrogen bonds with S190 and/or R119 (Fig. 5b, dotted lines).

Gypsum-DL also protonated one of the 2-aminopteridin-4-ol nitrogen atoms. While unusual, the resulting positive charge enables electrostatic interactions with D97 and may further strengthen the π-π stacking with W187 and Y101 by adding a cation-π interaction. Gypsum-DL’s protonated secondary amine may also form cation-π interactions with W187 and Y76 (Fig. 5b). It is admittedly unclear to what extent the Gypsum-DL enol tautomer and protonated amines are physiologically relevant, but these states may have contributed to the improved pose prediction in this case.

### Sample libraries for download

We used Gypsum-DL to process two small-molecule libraries. Both are available free of charge from http://durrantlab.com/gypsum-dl/ for use in VS projects. We first obtained a copy of the NCI Diversity Set VI in flat SDF format from the National Cancer Institute (NCI). This library includes compounds that have been carefully selected so as to have diverse pharmacophores and favorable chemical properties (e.g., high molecular rigidity, few rotatable bonds, limited chiral centers, etc.). The NCI provides relatively pure samples (≥ 90% by LC/Mass Spectrometry) free of charge upon request. Gypsum-DL produced 5996 3D models from 1558 input NCI structures (pH 7.4, 3.8 models per input molecule). Eight bridged compounds could not be processed. We confirmed that alternate ionization, tautomeric, isomeric, and ring-conformer forms were among the output structures.

We also processed a large library of 56,608 N-acylated unnatural amino acids. We first generated 2D representations of these molecules by reacting alkyl-halide, Michael-acceptor, and carboxylic-acid building blocks in silico, using chemical reactions developed by the Distributed Drug Discovery (D3) initiative [15–18, 34]. D3 is an educational program started at Indiana University–Purdue University Indianapolis in 2003. Its well-documented, combinatorial solid-phase synthetic procedures enable students—including undergraduates—to synthesize diverse compounds in a classroom-laboratory setting. Candidate ligands identified in VS of these compounds can thus be easily synthesized and experimentally tested. Gypsum-DL successfully processed all 56,608 input compounds, producing 148,240 3D models (pH 7.4, 2.6 models per input molecule).

To avoid amide-iminol tautomerization, we intentionally instructed Gypsum-DL to skip tautomer enumeration for the D3 compound set. The iminol form is rare in solution, though it is occasionally chemically relevant [35]. It is reasonable to consider the iminol tautomer if virtual-library compounds contain only occasional amide moieties (e.g., the NCI set). But every compound in the D3 library contains an amide moiety. Modelling the iminol tautomer would have needlessly expanded the library’s size, adding to the computational cost of any subsequent VS.

### Comparison with other programs

**Fig. 6:**
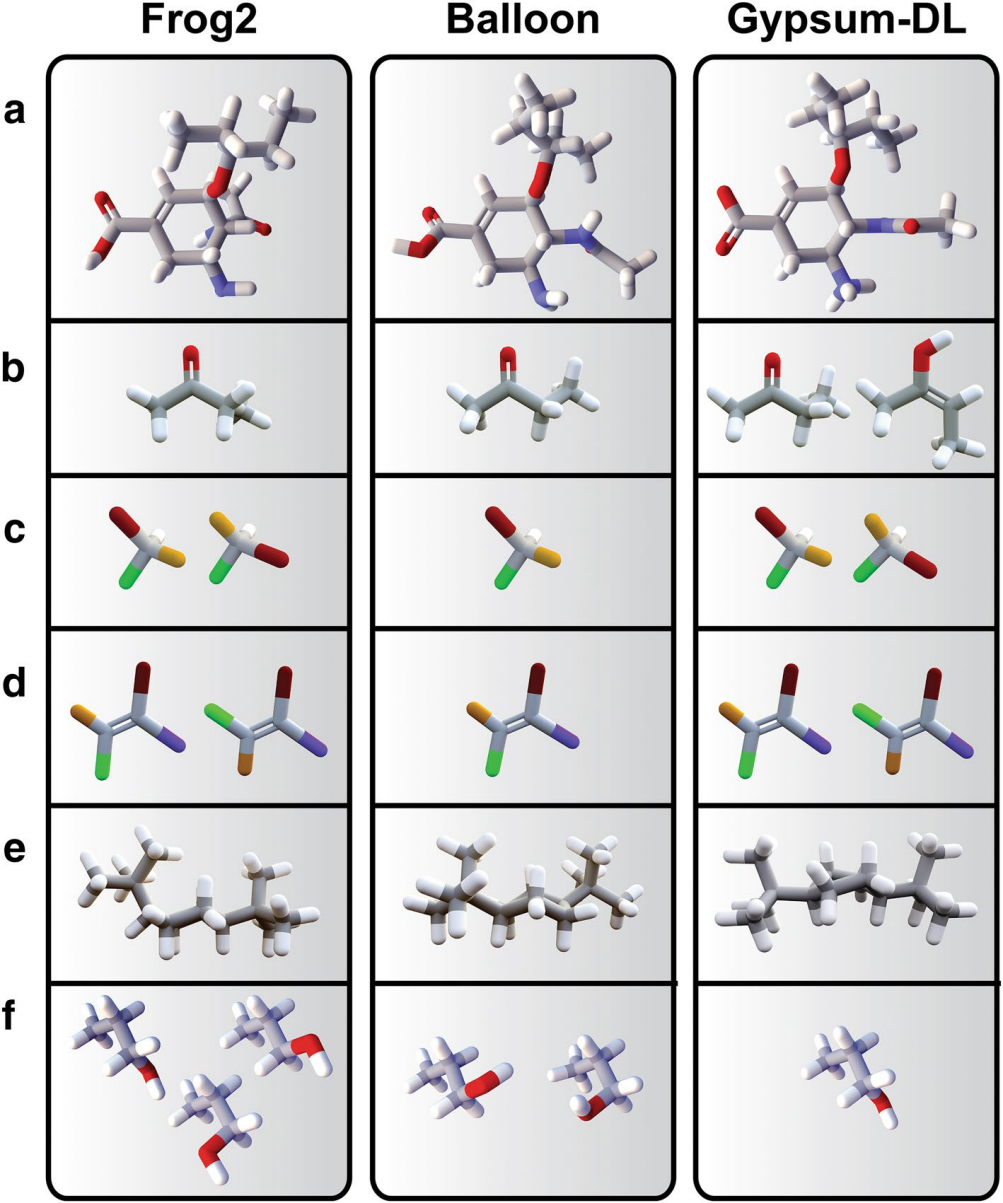
Example program output. Gypsum-DL outputs **a** deprotonated oseltamivir carboxylate, **b** both the ketone and enol forms of butan-2-one, **c** both the (*R*) and (*S*) enantiomers of bromochlorofluoroiodomethane, **d** both the *E* and *Z* isomers of 1-bromo-2-chloro-2-fluoro-1-iodoethene, **e** the twist-boat conformation of *cis*-1,4-di-*tert*-butylcyclohexane, and **f** only one rotomer of propan-1-ol. Image generated using BlendMol [33]

*Frog2* [5]* is another open-source program for preparing small-molecule libraries*. Its easy-to-use web interface is among its many strengths. This web-based approach arguably makes Frog2 more user friendly than Gypsum-DL. However, Gypsum-DL does offer some key capabilities that Frog2 lacks (Table 2). For example, Frog2 uses the Open Babel cheminformatics toolkit [10] to add hydrogen atoms to input molecules, but it does not consider alternate ionization states per a user-specified pH range. In contrast, Gypsum-DL uses the Dimorphite-DL algorithm [11] to predict ionization states. To illustrate the usefulness of this feature, we submitted oseltamivir carboxylate, an influenza neuraminidase inhibitor, to the Frog2 (v2.14) server (Fig. 6a). Frog2 protonated the carboxylic-acid moiety, despite the fact that it is largely deprotonated at physiological pH. In contrast, Gypsum-DL appropriately deprotonated the carboxylate group. Deprotonation is critical in this case, as neuraminidase-oseltamivir binding is governed largely by arginine-carboxylate electrostatic interactions that require a charged (deprotonated) carboxylate moiety [36].

**Table 2:** The available features of several stand-alone programs for converting molecular representations into 3D models

Program	Ionize	Tautomers	Chiral	cis/trans	Rings	Optimize	Free	Open Source	Web
Gypsum-DL	✓	✓	✓	✓	✓	✓	✓	✓	
OpenEye	✓	✓	✓	✓	✓	✓			
LigPrep	✓	✓	✓	✓	✓	✓			
Frog2			✓	✓		✓	✓	✓	✓
Balloon			✓	✓	✓	✓	✓		

OpenEye’s OMEGA/QUACPAC and Schrödinger’s LigPrep are commercial programs mentioned in the Introduction. Frog2 and Balloon are freely available

Frog2 is similarly limited in its ability to generate alternate tautomeric forms. To illustrate, we submitted butan-2-one, a ketone, to the Frog2 server (Fig. 6b). The server correctly returned a 3D model of the ketone, but it did not identify the alternate enol form, but-2-en-2-ol. In contrast, both the keto and enol form were present among the Gypsum-DL-generated 3D models.

Both Frog2 and Gypsum-DL performed comparably at enumerating unspecified chiral centers and cis/trans isomers. Both generated (*R*) and (*S*) enantiomers of bromochlorofluoroiodomethane when the input SMILES did not specify chirality (Fig. 6c). And both generated the *E* and *Z* isomers of 1-bromo-2-chloro-2-fluoro-1-iodoethene when given an ambiguous SMILES as input (Fig. 6d).

Gypsum-DL takes a more thorough albeit computationally expensive approach when generating alternate non-aromatic-ring conformations. Frog2 initially uses DG-AMMOS [37] to generate ring conformations, but the algorithm ultimately maintains rings rigid and considers only dihedral variations [5]. In contrast, Gypsum-DL uses geometry optimization and clustering to identify distinct ring conformations. To illustrate the advantages of the Gypsum-DL approach, consider *cis*-1,4-di-*tert*-butylcyclohexane (Fig. 6e). In the twist-boat conformation, both of the *tert*-butyl groups assume equatorial positions. The energy difference between the chair and twist-boat conformations of this compound are thus unusually small [38]. Frog2 generated models in only the chair conformation. Gypsum-DL generated models in the more stable twist-boat conformation.

An additional Gypsum-DL feature is advantageous in some VS contexts. Recall that many docking programs sample non-ring, single-bond torsions during the docking process itself. It is therefore computationally inefficient to dock otherwise identical models that differ only in their non-ring torsions. Frog2 generates these redundant models, but Gypsum-DL does not. As an illustration, consider propan-1-ol (Fig. 6f). Frog2 generated three redundant conformational isomers of propan-1-ol. In contrast, Gypsum-DL generated only one. We recommend Frog2 in those cases that require a more complete torsion library (e.g., 3D- or 4D-QSAR [39], pharmacophore modelling [40], etc.). Gypsum-DL is arguably better suited for use with flexible-ligand docking programs such as AutoDock Vina [12].

*Balloon* [6, 7]* is a free command-line program that targets more advanced users*. We tested Balloon on the Linux platform only, as we could not run any of the provided binaries on macOS Mojave. Balloon 1.6.7 is in many ways similar to Frog2 (Table 2). We applied the program to the same test molecules described above. Like Frog2, Balloon does not consider alternate ionization or tautomeric states (e.g., it protonated the oseltamivir carboxylate group and failed to identify the enol form of butan-2-one; Fig. 6a, b). While Balloon’s ring-generation algorithm produced the correct twist-boat conformation of *cis*-1,4-di-*tert*-butylcyclohexane (Fig. 6e), the *tert*-butyl moieties were not quite as equatorial as those of the Gypsum-DL model. Balloon also tends to generate redundant conformational isomers (e.g., it produced two propan-1-ol models; Fig. 6f). On the other hand, like Gypsum-DL and Frog2, Balloon does successfully enumerate unspecified chiral centers (e.g., bromochlorofluoroiodomethane; Fig. 6c) and cis/trans isomers (e.g., 1-bromo-2-chloro-2-fluoro-1-iodoethene; Fig. 6d).

*Free and open-source cheminformatics toolkits such as RDKit and Open Babe*l [10]* target the most advanced users*. These toolkits provide building blocks that programmers can assemble into more complex cheminformatics workflows. The RDKit and Open-Babel Python bindings are particularly useful for this purpose. Gypsum-DL is built on RDKit and RDKit-powered software (Dimorphite-DL 1.0 [11] and MolVS 0.1.1).

We built Gypsum-DL on RDKit, MolVS, and Dimorphite-DL rather than Open Babel in part because these packages have more permissive software licenses (BSD, MIT, and Apache version 2, respectively). Permissive licenses encourage broad adoption by allowing users to incorporate software into their own projects without having to adopt the same license. In contrast, Open Babel is released under a copyleft license (GNU General Public License, version 2), which requires that any derivate works also be copyleft. We note also that Gypsum-DL’s use of the Dimorphite-DL algorithm has several advantages over Open Babel’s approach (see Ref. [11] for details).

## Conclusion

Given the role that structure-based VS plays in modern drug discovery, effective techniques for generating 3D small-molecule structures are critical. Gypsum-DL is a free, open-source program that performs this important conversion to 3D. To minimize computational costs without sacrificing accuracy, we have designed Gypsum-DL to be highly parallel and computationally efficient. It can be easily incorporated into cheminformatic and drug-discovery workflows. Gypsum-DL’s easy-to-use command-line interface and default parameters make it accessible to intermediate users. Additional functionality and customizability allow advanced users to control more nuanced program parameters such as the thoroughness of conformer sampling, the pH range, and the output-file format.

Though a powerful tool, Gypsum-DL does have its limitations. For example, it often fails to identify low-energy conformations for large macrocycles. Gypsum-DL uses the ETKDG algorithm [20] to generate initial 3D models for subsequent UFF-based geometry optimization. ETKDG assigns macrocycle torsions based on acyclic-bond torsion patterns derived from experiment. We expect that future versions of the ETKDG algorithm will assign macrocycle torsions using the proper experimentally derived macrocycle torsion patterns. In the meantime, Gypsum-DL still generates valid, geometry-optimized macrocycle models, though the output conformations sometimes differ substantially from the most energetically favorable minima.

Future efforts will also include building a graphical user interface and/or web application to better accommodate the needs of researchers who are less familiar with the command line. We also hope to enable Windows multiprocessing in a future release. These current limitations aside, we believe Gypsum-DL will be a useful tool for researchers interested in structure-based, computer-aided drug discovery. A copy can be downloaded free of charge from http://durrantlab.com/gypsum-dl/.

## Availability and requirements

Project name: Gypsum-DL.

Project home page: http://durrantlab.com/gypsum-dl/.

Operating systems: Windows, macOS, Linux.

Programming language: Python 2/3.

Other requirements: RDKit, NumPy, SciPy, Mpi4py (optional).

License: Apache License, Version 2.0.

## Data Availability

Not applicable.
